# A mechanism for the cortical computation of hierarchical linguistic structure

**DOI:** 10.1371/journal.pbio.2000663

**Published:** 2017-03-02

**Authors:** Andrea E. Martin, Leonidas A. A. Doumas

**Affiliations:** 1 Department of Psychology, School of Philosophy, Psychology, and Language Sciences, University of Edinburgh, Edinburgh, United Kingdom; 2 Department of Psychology of Language, Max Planck Institute for Psycholinguistics, Nijmegen, The Netherlands; New York University, United States of America

## Abstract

Biological systems often detect species-specific signals in the environment. In humans, speech and language are species-specific signals of fundamental biological importance. To detect the linguistic signal, human brains must form hierarchical representations from a sequence of perceptual inputs distributed in time. What mechanism underlies this ability? One hypothesis is that the brain repurposed an available neurobiological mechanism when hierarchical linguistic representation became an efficient solution to a computational problem posed to the organism. Under such an account, a single mechanism must have the capacity to perform multiple, functionally related computations, e.g., detect the linguistic signal *and* perform other cognitive functions, while, ideally, oscillating like the human brain. We show that a computational model of analogy, built for an entirely different purpose—learning relational reasoning—processes sentences, represents their meaning, and, crucially, exhibits oscillatory activation patterns resembling cortical signals elicited by the same stimuli. Such redundancy in the cortical and machine signals is indicative of formal and mechanistic alignment between representational structure building and “cortical” oscillations. By inductive inference, this synergy suggests that the cortical signal reflects structure generation, just as the machine signal does. A single mechanism—using time to encode information across a layered network—generates the kind of (de)compositional representational hierarchy that is crucial for human language and offers a mechanistic linking hypothesis between linguistic representation and cortical computation.

## Author summary

Human language is a fundamental biological signal with computational properties that differ from other perception-action systems: hierarchical relationships between sounds, words, phrases, and sentences and the unbounded ability to combine smaller units into larger ones, resulting in a "discrete infinity" of expressions. These properties have long made language hard to account for from a biological systems perspective and within models of cognition. We argue that a single computational mechanism—using time to encode hierarchy—can satisfy the computational requirements of language, in addition to those of other cognitive functions. We show that a well-supported neural network model of analogy oscillates like the human brain while processing sentences. Despite being built for an entirely different purpose (learning relational concepts), the model processes hierarchical representations of sentences and exhibits oscillatory patterns of activation that closely resemble the human cortical response to the same stimuli. From the model, we derive an explicit computational mechanism for how the human brain could convert perceptual features into hierarchical representations across multiple timescales, providing a linking hypothesis between linguistic and cortical computation. Our results suggest a formal and mechanistic alignment between representational structure building and cortical oscillations that has broad implications for discovering the computational first principles of cognition in the human brain.

## Introduction

Detecting relevant signals in the environment is a crucial function in biological systems. For humans, language is a critical, if not the defining, species-specific environmental signal to detect. As such, it is not surprising that the human auditory system is specialised for speech processing [e.g., 1,2]. However, very little is known about the biological mechanisms that detect the “linguistic signal” within speech (i.e., words, phrases, sentences, meaning), apart from the fact that that cortical entrainment to the acoustic envelope of speech likely plays a fundamental role in spoken language comprehension [3,4,5]. In this paper, we show that using time to encode information about the structural relationship between representations within the linguistic signal, or *time-based binding*, can generate the kinds of representations that can support human language within a layered neural network and produces oscillations that are highly similar to human cortical signals. By inductive inference, time-based binding, or a formal equivalent, can support language-related cortical computation.

In order to detect linguistic signals in the environment, the human brain requires a computational system that can generate hierarchical representations from the sequential perceptual input of speech or text. Generating abstract, higher-level representations like sentences from either sensory source is likely to rely on simultaneous tracking of multiple levels of the linguistic signal [e.g., 3,6,7,8,9]. However, operating over these multiple levels of signal must be a mechanism that can compute structured, discrete representations from unstructured continuous input in time—essentially, an operation that can form representations from feature sets, as in other areas of perception [10,11,12,13]. However, complications arise because human language has computational properties that set it apart from other domains of cognition and perception, namely, representation of discrete infinity, arbitrary form-meaning correspondence, and learnability constraints [12,14,15,16]. One important property is *compositionality*—when interpreting a simple sentence like *Fun games waste time*, being a proposition in human language requires that the representation of *fun* as a discrete word is maintained, even after computation of the phrases *Fun games*, *Fun games waste*, and the sentence *Fun games waste time*. To make things more complex, this stringent representational requirement holds below, and beyond, the word-level: both low-level (e.g., phonetic, syllabic, and orthographic) and high-level (e.g., phrases, sentences, event-structures, discourse) representations have discrete hierarchy in the face of compositionality. Linguistic computation in the human brain, therefore, must generate a representational hierarchy that can represent the compositional product of input representations while maintaining discrete input units [17,18].

Such a representational hierarchy suggests that a first principle of linguistic computation is a form of relationality: the system must determine whether (or not) to relate and (then compose) input representations with one another. One way to achieve compositionality without sacrificing information is to represent the relationship between input and output representations explicitly [18,19]. In a basic sense, this computational situation is akin to that which the system faces during analogical or relational reasoning, in which the relation between representations must be computed. For example, when the system must discriminate the conceptual propositions "John loves Mary" from "Mary loves John," it must discriminate sequences of identical input arguments that have very different consequents by virtue of their relational structure [20,21]. Thus, it appears that the encoding of relational structure is a powerful tool for a biological system that solves the problems that human cognition evolved in response to. Moreover, from a biological systems perspective, it is highly desirable for a single (cortical, computational) mechanism to have the capacity to compute over multiple domains, or to function as a subroutine in multiple cognitive functions [11,22,23,24]. Below, we describe the computational challenge of (de)compositionality and describe a mechanism that can encode and preserve hierarchical relational structures from unstructured input within a layered neural network. Our approach contrasts with extant associative models of speech and language processing, cognition, and cortical microcircuits.

If you can understand the sentence *Fun games waste time*, you typically also know that the verb *waste* can combine with phrases other than *fun games* and nouns other than *time* without changing the meaning or syntactic function of *waste*. One way to achieve this functionality is to represent *waste* independently from *fun games* and *time* while also generating representations of the (grammatical) relations between the inputs *fun*, *games*, *waste*, and *time*. Those representations of grammatical relations can then be generatively applied to other inputs. A linear form of such a representation would be something like:

$${\left\{{\left\{{\text{fun}}_{\text{adj}}{\left\{{\text{games}}_{\text{n}}\right\}}_{\text{NP}}\right\}}_{\text{ADJP}}{\left\{{\text{waste}}_{\text{v}}{\left\{{\text{time}}_{\text{n}}\right\}}_{\text{NP}}\right\}}_{\text{VP}}\right\}}_{\text{IP}}$$ (1)

where the most distal level of brackets codes the whole sentence representation, and another set of brackets codes the adjective, noun, and verb phrases. The curly brackets in Eq (1) indicate phrasal grouping in an approximation of formal linguistic notation, where IP = inflectional phrase or sentence, AdjP = adjective phrase, NP = noun phrase, VP = verb phrase, and adj = adjective, n = noun, v = verb. *Fun games* is an adjective phrase that contains the adjective *fun* and the noun phrase that contains the noun *games*. That adjective phrase is composed with a verb phrase, which, itself, is the product of combing the verb *waste* with the noun phrase containing the noun *time*.

In contrast, systems that do not encode relational structures in inputs, such as traditional recurrent neural networks (RNNs), would represent *fun games waste time* by creating conjunctive representation, such that {*fun*} and {*games*} becomes the holistic {*fun games*}, losing any internal distinction between {*fun*} and {*games*}. The end product representation would be something like {*fun games waste time*}, with no subunit of the sentence being independently represented. In such a system, {*fun games waste time*} would bear no relation to {*games waste time*} or {*games are fun*} nor to {*long showers waste water*} because *waste* is not represented independently [25,26]. More generally, systems that use tensor products (the outer product of two vectors or matrices) to represent or bind stimuli together in the network suffer from the same problem in that once you bind the two things together in the network, you cannot separate them anymore. This pitfall stems from the fact that there are multiple solutions to decomposing a tensor into its input vectors—you can never know which solution is "the right one" for the original input vectors once you have multiplied them. That means you cannot compose and decompose representations in a tensor system without losing information, which is unsuitable for linguistic representation and for any neural or biological system that must be both compositional and hierarchical [20].

To circumvent this problem, relational structures can be encoded via *binding* [13,27]. We define binding for our purposes here as the representational state where two codes in the network are linked together for processing but where their representations are not defined by this particular instance of binding. Such a mechanism allows the system to both maintain independent representations of input units while also composing the same representations together during processing, as needed. In sum, our focus here is on the representation of sequences in which implicit ordinal and time-sensitive relationships matter, and, in fact, may signal the hierarchical relationships that have been compressed into that sequence. The basic idea is to encode the elements that are bound in lower layers of a hierarchy directly from the sequential input and then use slower dynamics to accumulate evidence for relations at higher levels of the hierarchy. This necessarily entails a memory of the ordinal relationships that, computationally, requires higher-level representations to integrate or bind lower-level representations over time—with more protracted activity. This temporal binding mandates an asynchrony of representation between hierarchical levels of representation in order to maintain distinct, separable representations despite binding.

### Time-based binding in a layered neural network

Here we describe how representations that maintain relational structure can be composed and decomposed in a layered neural network. A simple computational mechanism, *time-based binding*, in which time is used to carry information about the relationships between representations in the input, is one way to achieve such an architecture. It is a truism and/or a biological principle of cortical organisation that "neurons that fire together, wire together" [28]. As such, some time-based binding systems use synchrony of firing to link representations together in the network for processing [e.g., 29]. Conversely, neurons that do not fire in synchrony can stay independent, and the proximity in time between firings can be exploited to carry information. Discovery of Relations by Analogy (DORA; a symbolic-connectionist model of relational reasoning; the full computational specifics of the model can be found in Doumas et. al [19], operating procedure available in Appendix A) exploits the synchrony principle in order to keep representations separable in the limit while binding them together for processing. This situation means that the system can be said to have *variable-value independence* when the representation of a given variable and its particular value at a moment in time are explicitly, independently represented [20,30]. A representation of a variable and its value must be able to function separately in any system performing a computation that requires relationality or (de)compositionality, such as during analogical reasoning, language processing, and likely many other higher-level cognition functions [31]. In a system with variable-value independence, statistics about the association between representations can still play an important role, but those statistics are not the sole basis of the representational architecture. In other words, variable-value independence allows the system to represent a variable, its value, and also to compute statistics about their association without changing the core representations.

**Fig 1:**
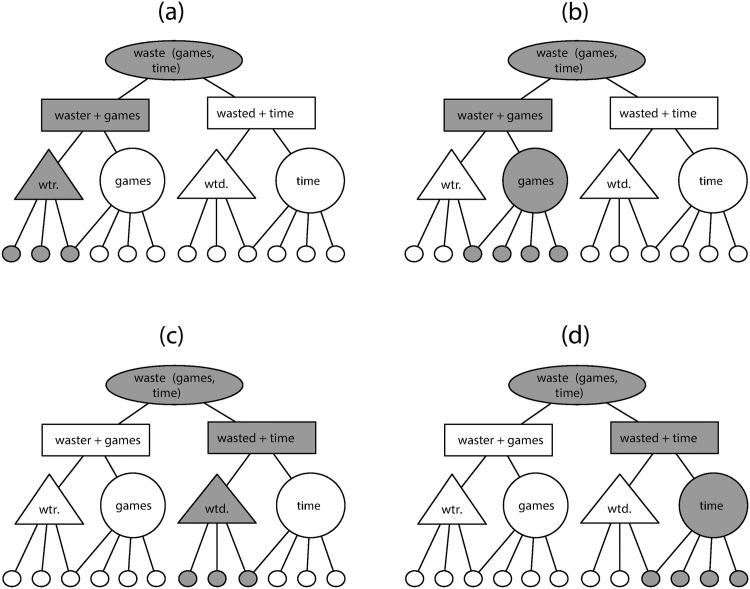
A DORA representation of the proposition *waste* (games, time) during processing that illustrates how time-based binding works. We use different shapes to represent units in different layers (ovals for Proposition node "P-units"/sentences, rectangles for Role-filler binding nodes or "RB units"/phrases, triangles and large circles for Propositional Object (PO) units/words and argument roles, and small circles for semantic units/features) for the purposes of clarity. Abbreviations "wtr." and "wtd." signify the role of *waster* and *wasted* in the proposition *waste*(games, time), respectively. In the model, these units are simply nodes in different layers of the network. Darker units denote when a unit is firing at a given time step (panels a–d in the Fig 1), which in this case corresponds to 250 msec/4 Hz. Please see page 18 for a detailed discussion of P-units, RB-units, and PO units.

In brief, DORA's primary computational assumptions are (1) a neural network with layers of units, (2) lateral inhibition, (3) separate banks of units, (4) Hebbian learning, and (5) sensitivity to time. The layered structure of the network, combined with sensitivity to time as carrying information about the relations between the nodes in the layers of the network, is one solution to preserving the structure in (1). After learning (please see Appendix A in [19] and pp. 8–13, 16–21 in the main text), the network represents words, phrases, and sentences across layers of the network, such that words and brackets in (1) correspond to nodes on different layers of the network. Nodes on higher layers code for the composition (a phrase) of two sublayer nodes (words) and fire when either of the subnodes below it fire in time. Asynchrony of unit firing (in this case, of a node, but in theory, of a neural population or assembly, see [32]) allows the network to bind representations together for processing on a higher layer while maintaining independent codes for the input representations on a lower layer of the network. The combination of layers and time-based binding via asynchrony is what allows the network to have (de)compositionality—representations of compositional product *and* the decomposed inputs on different layers of the network. To represent (1), the network encodes the adjective phrase {fun_adj_{games_n_}_NP_}_ADJP_ over two layers of the network; on the lower layer, one node codes for the word {fun_adj_} and another for the word {games_n_}(see Fig 1). On the next layer above that, the phrasal node will activate when the nodes {fun_adj_} and {games_n_} fire. These word nodes fire staggered in time, or at an asynchrony, and still activate the node that codes for the phrase {fun_adj_{games_n_}_NP_}_ADJP_. A similar configuration codes the phrasal binding between {waste_v_} and {time_n_} (a node that codes the verb phrase {waste_v_{time_n_}_NP_}_VP_). The relationality between the phrases {fun_adj_{games_n_}_NP_}_ADJP_ and {waste_v_{time_n_}_NP_}_VP_ is represented by encoding information about being an agent (e.g., "the waster") or a patient (e.g., "the wasted") in a two-argument predicate. One way to represent predicate argument relationships in a neural network is to code role information in a separate node from the particular argument that fills that role at a given processing moment. When the role slot in a predicate is represented separately from the given input, predicate–argument relationships can be generatively applied to any input that predicate is associated with in the dataset (see Fig 1). These argument role nodes, which code for the role-filler binding relation, can be learned in the same way the word nodes are (see [19] for details). Lastly, a sentence node that represents the relation between {fun_adj_{games_n_}_NP_}_ADJP_ and {waste_v_{time_n_}_NP_}_VP_ as a sentence (a node that fires when the AdjP and VP units fire, and thus codes for the whole sentence {{fun_adj_{games_n_}_NP_}_ADJP_{waste_v_{time_n_}_NP_}_VP_}_IP_) is on the highest layer. The layered structure of the network is a core assumption and is necessary for time-based binding to function and for predicates to be represented in a connectionist network. We base the assumption that the system has layers on the broadest notion of cortical organisation; however, the representational codes for a given layer are learned from the input (see [19] for detailed explanation of the learning process, which itself is not central to the results reported here, nor to the theoretical claim made here). The main advantage of time-based binding is that it avoids the superposition problem that a system that only uses synchronous firing alone would face.

In sum, the essential difference between DORA and other connectionist models is that DORA binds representations using asynchrony of unit firing and thus can vary the level of representation at which the asynchrony is maintained. Slight asynchrony in unit firing leads to independent, discriminable sequences of representation across layers. Units on the next layer of the network then code for or fire when two or more subunits fire within a certain time of each other, which results in a hierarchical representation that can discriminate sequences with the same inputs and represents input values independently. In this way, DORA learns structured representations of relations from unstructured (holistic flat feature vector) inputs and is based on traditional connectionist computing principles (i.e., layers of interconnected nodes passing activation via weighted connections that are modified via Hebbian learning). However, in contrast with most connectionist networks, DORA effectively learns and implements hierarchical symbolic representations [20,33]. As summarised above, DORA does this by using time to encode relational information and uses comparisons of distribution of activity in time to subset units into function representations and to continue refining them throughout the learning process [19]. DORA accounts for numerous phenomena from relational learning, as well as its development (e.g., [19,34,35,36,37]).

### Tracking hierarchical representations in language processing

Although there are seldom clear physical boundaries in speech input that directly correspond to higher-level representations (i.e., phonemes, words, phrases, and sentences), we perceive and experience complex discrete representations in continuous input. A growing body of evidence suggests that this perception is based in the entrainment of cortical brain rhythms to speech [3,5,6,7]. While such neuroimaging evidence is suggestive and its implications tantalising, there is a mechanistic gap between such cortical signals and the representational output of the system (as described by formal theories, or as might be formalised in any computational system). An adequate mechanistic linking hypothesis between linguistic and cortical computation would explain how the system goes from input of perceptual features to a hierarchy of structured representations and would describe *how* the computational mechanism gives rise to the observed cortical activation states. Furthermore, such a hypothesis would shed light on whether the observed cortical signal reflected “mere” tracking of hierarchical linguistic representations or whether it, in fact, reflects the online generation of hierarchical linguistic structure.

To carve the computational problem at its joints, we turn to Ding et al. [6], who report cortical tracking of hierarchical linguistic structures (i.e., words, phrases, and sentences) in oscillatory data in both electrocorticography and magnetoencephalography recordings. Ding et al. presented auditory strings of synthesised speech in Mandarin Chinese and American English. Their stimuli were isochronous but manipulated the structural relationship between the syllables such that, in one condition, there was no meaningful relationship between the string of syllables/words (“*walk egg nine house”*), in a second condition, phrases were formed from adjacent syllables (*“flat table angry birds”*), and in a third condition, sentences emerged (*“new plans gave hope”*). An increase in power at frequencies in the oscillatory response on the timescale of syllabic/lexical rate (4 Hz), phrasal rate (2 Hz), and sentential rate (1 Hz) tracked the hierarchy of linguistic representation. Importantly, Ding et al. showed that the signal could not be attributed to entrainment to acoustic information, transitional probability, or word predictability [6]. Finding cortical entrainment at these slower oscillations (1 Hz, 2 Hz) suggests that cortical populations are entraining to abstract linguistic representations like phrases and sentences. This is remarkable because it is unclear what, if any physical instantiation of these higher-level stimuli are present in the speech signal—at least, there is no (known) set of acoustic cues in speech that reliably or diagnostically signal word, phrase, and sentence structures. That Ding et al. [6] found cortical entrainment to such higher-level linguistic structures suggests that the brain is, nonetheless, sensitive to the presence of these abstract linguistic representations.

Strikingly, DORA predicts such a representational pattern in oscillatory unit firing. DORA is a model of how information is represented—consequently, the simulations we present here are in no way intended as a fully articulated model of parsing. Furthermore, we want to emphasise that DORA represents a form of role-filler binding predicate calculus, in which expressions can be, but are not always, nor even usually, formally equivalent to the natural language expression. Future work is needed to derive representations in DORA with one-to-one correspondence to natural language; however, for the sentence stimuli tested herein, the difference between natural language form of the expression and the DORAese predicate logic does not bear on the theoretical conclusion that we draw from the results of our simulations. However, if the structure of information in DORA turned out to resemble how language appears to be represented in the human brain, that finding would indicate mechanistic synergy between the two computational systems.

We present simulations of sentence processing within a model of relational concept learning that provides a mechanistic explanation for how representational structure emerges from unstructured input. We show that the model not only represents sentences and exhibits oscillatory activation patterns that are strikingly similar to human cortical oscillatory brain activity during exposure to the same stimuli [6]. We further demonstrate that the model, as a result of processing the sentence stimuli, forms representations of sentence meaning that reliably discriminate between semantically composable grammatical sentences and syntactically intact but semantically non-composable sentences. Our results, though not a model of parsing nor of a cortical microcircuit, are an existence proof that using time to carry information (“time-based binding,” or generating explicit hierarchical representations by encoding structural relations in time) addresses two hard problems for cognition that have so far been investigated entirely separately: sentence processing and analogy (see [19] for human-level simulations of relational and analogical reasoning). In order to determine if the oscillatory pattern from Ding et al. [6] can arise from the serial presentation of a stimulus at 4 Hz alone, we ran the same simulations in a RNN. The RNN simulations test whether signals like the cortical oscillations observed by Ding et al. [6] can arise from a system without time-based binding or representational hierarchy, in which they might arise from seriality alone.

### The current study

**Fig 2:**
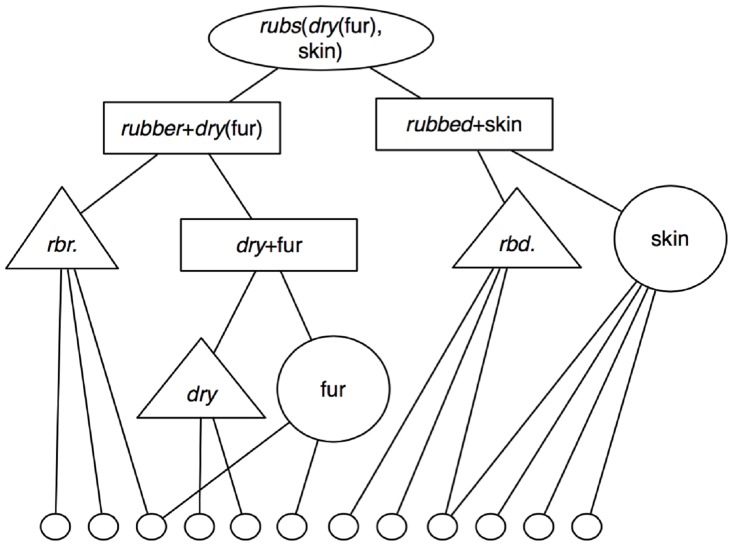
A representation of the sentence "Dry fur rubs skin" in Learning and Inference with Schemas and Analogies (LISA; [21]) /DORAese predicate calculus. We use different shapes to represent units in different layers (ovals for P-units/sentences, rectangles for RB units/phrases, triangles and large circles for PO units/words, and small circles for semantic units/features) for the purpose of clarity. In the model, these units are simply nodes in different layers of the network.

DORA processed the English sentence stimuli from Ding et al. [6], as well as three control conditions wherein either only syntactic relationships, but no compositional meaning, were present (*Jabberwocky condition*), no syntactic relationships existed between the words in the input (*Word List condition*), or only phrases existed (a version of the phrase-only condition from Ding et al., *Phrases condition;* please see [6] for the list of Grammatical Stimuli and S1 Text for a list of our additional stimuli and Fig 2 for a schematic of Grammatical sentence representations in DORA's predicate calculus). We observed whether DORA represented the phrases or sentences correctly, and recorded the oscillatory pattern of unit firing in layers of DORA’s network during processing. Finally, in contrast to available brain data, we assessed the content of the representations DORA generated during processing of Grammatical and Jabberwocky sentences. We plotted the activation of existing predicates in memory in response to the hierarchical representations that parsing generated in those two conditions. To discount the hypothesis that the oscillatory pattern stems only from serialised processing, we repeated the four simulations in a RNN for comparison. Please see the Methods section for a description of the RNN that we trained. In brief, this was a standard RNN with one hidden layer that was trained on the same stimuli as in the DORA simulations.

## Results

**Fig 3:**
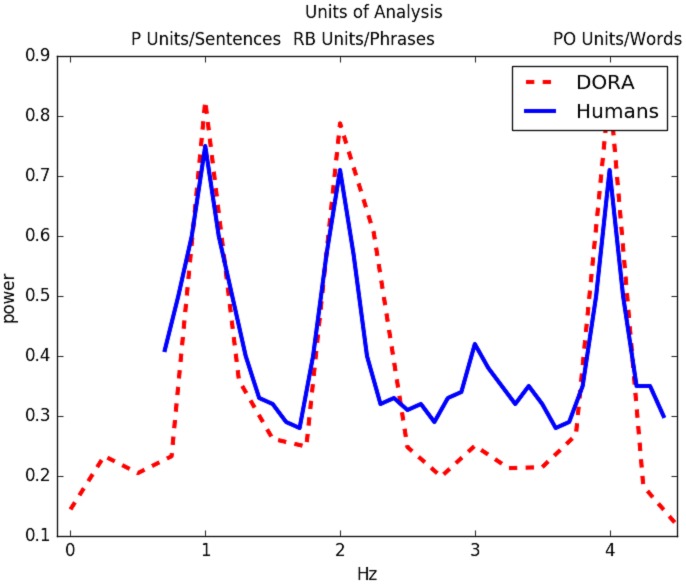
Grammatical sentences: DORA network power spectrum compared to human cortical oscillations. The solid line represents cortical power while participants listened to four syllable/word sentences played over 1 s in Ding et al. [6]. Power increases are evident at the 1 Hz (sentence duration), 2 Hz (phrase duration), and 4 Hz (word duration) range. The dashed line depicts firing in DORA while processing the same sentences used in Ding et al. Units in DORA fire for the duration of the sentence, at intervals of half the length of the sentence and at intervals lasting a quarter of the length of the sentence. Data from the stimulation and the code to run it are available at https://osf.io/eb2vp/ and https://github.com/AlexDoumas/dingetal_sent.

**Fig 4:**
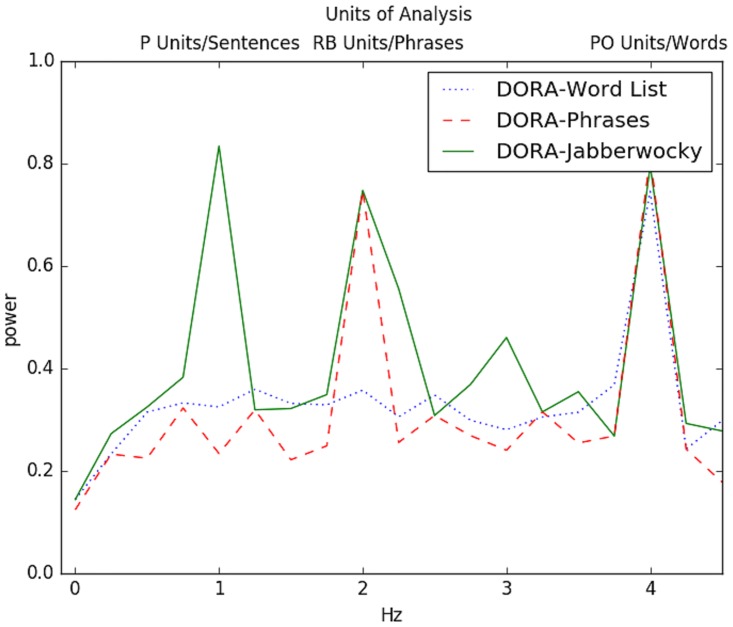
DORA network power spectrum plot of the Word List, Phrases, and Jabberwocky conditions. For Word List, an increase in firing only occurred at the 4 Hz range, corresponding to firing of nodes coding for words. Lack of firing at other frequencies indicates that no hierarchical representations were processed in the Word list condition. In the Phrases condition, there was an increase in power at 2 Hz and 4 Hz, indicating that units coding words and units coding phrases were active during the processing of this condition. No sentence units were active. In the Jabberwocky condition, there was an increase at 1, 2, and 4 Hz range, similar to the pattern seen for grammatical sentences, indicating that hierarchical representations were indeed activated. See Fig 5 for a comparison of activation across the propositions in the model's long-term memory between Jabberwocky and Grammatical sentences. Data from the simulations and the code to run them are available at https://osf.io/eb2vp/ and https://github.com/AlexDoumas/dingetal_sent.

**Fig 5:**
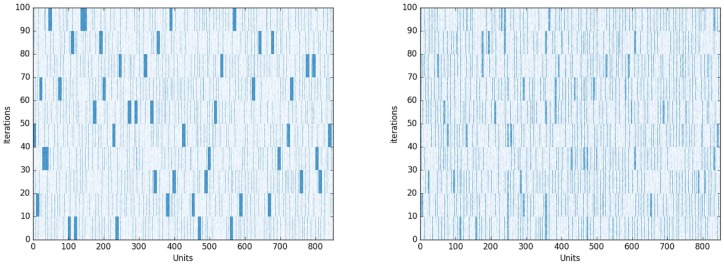
Left Panel: Grammatical. Plot of active propositions in memory across trials in the Grammatical condition. On the *x*-axis are P-Units, on the *y*-axis are processing iterations of the model, equivalent to trials or instances of processing a sentence during the simulation. The darker colour indicates more activation of existing propositional role-filler binding combinations in memory. Grammatical sentences resulted in stronger activation of extant propositions than Jabberwocky sentences did. Right Panel: Jabberwocky. The Jabberwocky condition did not activate as many single existing propositions in the model's memory as the Grammatical condition did, rather, activation was spread more broadly across memory, despite both conditions producing similar oscillations in DORA. Data from the simulations and the code to run them are available at https://osf.io/eb2vp/ and https://github.com/AlexDoumas/dingetal_sent.

The results of the simulations are presented in Figs 3, 4 and 5. Interestingly, the oscillatory pattern of firing in the various layers of DORA during processing of sentences, closely mirrored the patterns observed by Ding et al. [6]. Specifically, just like the cortical signals, for Grammatical sentences, DORA showed an activation burst that lasted throughout the processing of the sentence (i.e., firing in the 1 Hz range), activation bursts at twice the rate of the whole sentence burst (i.e., firing in the 2 Hz range), aligned with phrase-level processing, and activation bursts at four times the rate of the whole sentence burst (i.e., firing in the 4 Hz range), corresponding to word-level processing. However, the activity in the Word List condition also resembled the human data, in which in both cases, there was only spiking at 4 Hz but not at slower frequencies, which indicates that larger constituents were neither tracked nor formed in this condition. The Jabberwocky condition, which has no analogue in the available human data, resulted in oscillations that resembled the Grammatical condition, suggesting that the model is sensitive to something akin to syntactic structure or category during processing of Jabberwocky. Finally, we had the model process the Phrases condition, which contained strings of phrases, but not sentences, and it showed a power increase at 2 Hz and 4 Hz, resembling the human data to a phrase-only condition in Ding et al. [6].

To assess the nature of the representations DORA generated during parsing, we plotted activation maps of the contents of DORA’s memory over 100 trials (instances of processing a sentence in the simulation) in the Grammatical and Jabberwocky conditions. Note that the oscillatory firing pattern from the Word List condition indicated that DORA did not compose role-filler bindings to compare with memory in that condition. The difference in activation of existing representations in DORA’s memory between the Grammatical condition (Fig 3) and the Jabberwocky condition (Fig 4) are illustrated below (Fig 5). We plot only the activation of propositions that contain a word that was present in the stimuli in these particular trials (0–100). The darker bars indicate that the hierarchical representations formed during processing more strongly activated representations that were already in the semantic memory of the model. Jabberwocky sentences activated fewer existing token units and activated these units to a lesser extent than the Grammatical sentences did, suggesting the model is generating novel, syntactically licensed representations that are "representationally unusual," comparable to the human experience of reading syntactically intact but semantically anomalous Jabberwocky sentences. We performed a *t*-test comparing the number of units above threshold across the 100 runs in the Grammatical and Jabberwocky conditions and found that more units were active while processing a Grammatical sentence (mean units active = 70.78) than a Jabberwocky sentence (mean units active = 31.95); *t* = 58.312, *p* < 2.2e−16.

**Fig 6:**
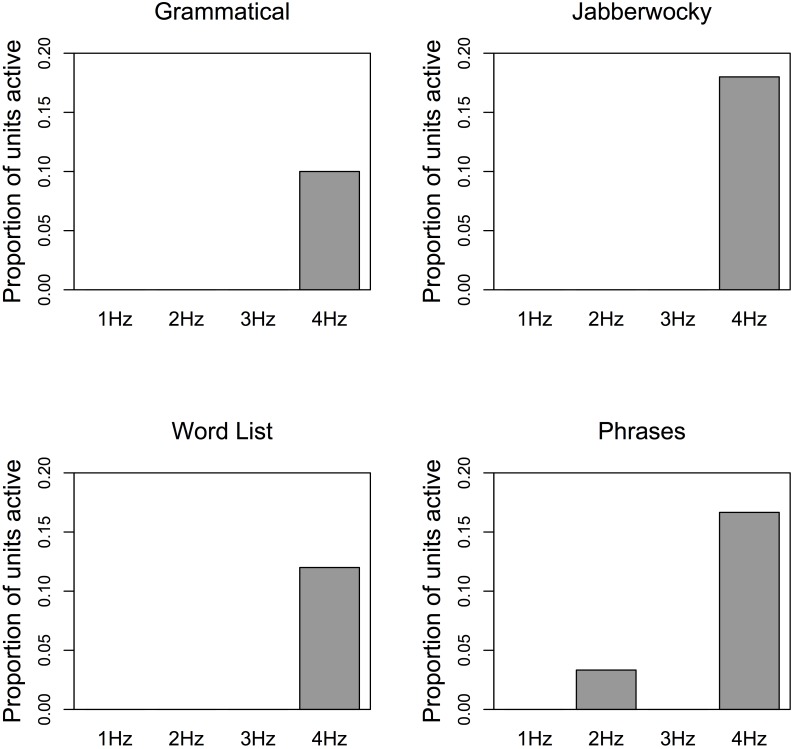
Proportion of units (range: 0–0.2) active above threshold (0.7) in the recurrent layer of the RNNs. Note: total number of units in the recurrent layer *n* = 50 for all conditions except for Phrases, in which *n* = 30. On average, between 5–9 units out of 50 in the hidden layer activated at 4 Hz. There was no evidence of activity at 1 Hz or 2 Hz, and hence no evidence for coding or tracking of linguistic structures in the RNN. Data from the simulations and the code to run the simulations are available at https://osf.io/eb2vp/ and https://github.com/AlexDoumas/dingetal_sent.

We then repeated the four simulations in an RNN. In order to determine the activation level of the RRNs at each frequency, we attempted to identify units in the trained RRNs output layer that were active above a threshold of 0.7 consistently at 1 Hz, 2 Hz, 3 Hz, and 4 Hz across all sentences in each condition (Fig 6). Activation in the hidden layer corresponds to representations of the statistical patterns that the network learns, so finding patterns here would indicate that the RNN learned hierarchical linguistic structures from the input. For the Grammatical condition, zero units in the hidden layer were active at the 1 Hz, 2 Hz, and 3 Hz rates; five units (out of 50 hidden units in the network) were active above the threshold at the 4 Hz rate. For the Jabberwocky condition, zero units were active at the 1 Hz, 2 Hz, and 3 Hz rates; nine units were active above the threshold at the 4 Hz rate. For the Word List condition, zero units were active at the 1 Hz, 2 Hz, and 3 Hz rates; six units were active above the threshold at the 4 Hz rate. For the Phrase condition, one unit was active at the 2 Hz rate; five hidden units out of 30 nodes were active above the threshold at the 4 Hz rate. Fig 6 shows the proportion of units (ranging from 0–0.2 proportion of units) active in the recurrent layer at each rate in each condition. We not that the RNN achieved perfect performance, that is, it learned the sentences such that for any input word, the RNN could tell you the next *n* words with 100% accuracy. The difference between the RNN and DORA is that, in doing so, the RNN did not do anything in a way that resembles what humans do, that is, it neither formed (symbolic) hierarchical representations, nor oscillated, nor oscillated in a way that resembles the cortical signals to the same stimuli.

## Discussion

We have presented simulations from a computational model that learns and generates hierarchical structure from an unstructured string of lexical input. The model, DORA [19], was built for a completely different purpose (learning relational concepts to perform analogical reasoning) but achieved the current outcome for sentence processing without any formal or structural changes from its original state. DORA learns and generates structured, symbolic representations using time-based binding in a layered neural network [19]; it is this computational architecture that enables the model to process sentences, to form hierarchical representations in general, and is what gives rise to the oscillatory pattern that resembled cortical oscillations. DORA is a model of how information is represented in the human mind, and perhaps more speculatively, how *information* might be macroscopically represented in cortical networks. It is not a model of parsing or cortical microcircuits. Nonetheless, when processing sentences like *fun games waste time*, the English sentence stimuli from Ding et al. [6], the oscillatory firing pattern of units in various layers of DORA closely resembled the cortical oscillations observed by Ding et al. [6]—an activation burst lasting throughout the processing of the sentence (1 Hz range), activation bursts at phrase-level processing, or twice the rate of the whole sentence burst (2 Hz range), and activation bursts at word-level processing, or around four times the rate of the whole sentence burst (4 Hz range). Furthermore, the representations that the model generated during parsing reliably reflected whether the sentence was grammatical, jabberwocky, a list of phrases, or a list of words. This result indicates that DORA's architecture generates representations that are both semantically rich and structurally sensitive, two properties that are essential to human language. In contrast, a traditional connectionist network (an RNN) failed to show an oscillatory pattern that resembled the cortical signal, but a few nodes coded words at 4 Hz. Thus, there was no evidence of the RNN representing hierarchical linguistic structures. This contrast showed that seriality of processing alone is insufficient to produce the oscillatory pattern observed in humans and in DORA.

Our results naturally beg the converse question, as to whether any system with representational hierarchy could produce the oscillatory pattern of activation that [6] and DORA show. For example, could natural language processing (NLP) parsers, which feature representational hierarchy and were developed to specifically parse natural language in a machine, produce oscillations and the pattern seen in [6] and in our simulations? In principle, any system of hierarchy that is (de)compositional has the representational ingredients to encode units that could be fired in a sequence. However, we would argue that any given representational hierarchy could only produce oscillations *if it were combined with time-based binding*, which, as far as we know, no NLP system features. In DORA, time-based binding *is* the oscillation of activity throughout the network, which is part of the reason why the RNN did not show oscillatory activity nor the specific pattern from [6]. The representational structure of DORA (a (de)compositional role-filler binding predicate logic) is what makes the oscillations take the form that the data from [6] have. In terms of the specifics of the observed 1-2-4 Hz pattern, both [6] and our simulations are highly shaped by the word presentation rate of 250 ms/4 Hz. But without time-based binding, there is no mechanism to produce oscillations in a network, even in a NLP parser or other system with representational hierarchy.

In sum, we remain relatively agnostic about the specific details of the required representational hierarchy because we do not yet know how to link the predicate calculus representations we use to natural language mental and cortical representations. What we are not agnostic about is the need for asynchronous time-based binding in order to produce oscillations in a neural network, as well as the need for representational hierarchy to produce the particular pattern of oscillations observed here and in [6].

Furthermore, another difference between DORA, RNNs, and NLP parsers is that DORA is a model of how information is represented and computed in the human mind. In other words, DORA makes a specific theoretical claim about how the human mind represents (some kinds of) information—that is, it does so in a *relational*, structured, hierarchical manner, which is realised via time-based binding. RNNs and NLP parsers do not make such theoretical claims about the architectures and mechanisms of mental and cortical computation.

In DORA, structured representations form during learning and become activated during later processing. Our simulations and theoretical claims concern the way in which information is activated during processing. However, we note that it is the same computational mechanism that can generate representations during learning, which also performs processing: the use of time to carry information about the relations between inputs, implemented as systematic temporal asynchrony of unit firing across layers of the network, which we have called "time-based binding." Through temporal asynchrony of firing, the model can use separable populations of units to maintain activation of different levels of representation as they occur in time. This ability to maintain hierarchy is the computational feature that turns out to be crucial for representing and processing the kinds of relations that are necessary to represent human language and is likely to be necessary for generating hierarchical levels of representation in any cortical network. We note here that our argument that temporal asynchrony is the mechanism that gives rise to hierarchical representation finds traction also in the conceptual terminology of neural oscillations, namely that temporal asynchrony corresponds to neural desynchronisation. Importantly, synchrony and asynchrony of unit firing in time are not orthogonal mechanisms; in fact, they are the same function or variable with different input values (e.g., sin(x) and sin(2x)) that can carry different information (see [38]). Our mechanistic claim is that it is *asynchrony* that allows the system to bind information for processing while maintaining (de)compositionality and generating hierarchical representations. Binding or forming representations through synchrony alone would fail at the superposition problem, effectively superimposing a variable and its value onto a single, undecomposable representation.

The generation of structured representations in DORA is a form of *predication* [19]. DORA’s representations are symbolic predicates that code for the invariant relations between features and objects (e.g., between the features “fun” and the object “games,” the feature “adjective” and the object “fun,” or the feature “noun” and the object “games”), or between objects and each other (e.g., between the object “fun games” and the object “waste” [19,39]). The resulting computational architecture thus explicitly represents invariant relations between input representations (or variables) and output representations (or values), as a function. This architecture is in contrast with RNNs and current deep-learning algorithms, which associate input and output via statistical association and, therefore, do not (currently) preserve compositionality or represent relational structures [20,25,26,30,40,41]. Because DORA explicitly codes invariance as a function, it can combine novel inputs with existing predicate structures, leading to productive, combinatorial generativity of representation that is neither maligned by violations of statistical regularity, and not reliant, in principle, although not demonstrated here, on hard coding of representations (cf. [42]). Though not directly relevant to the claims we make here, DORA is able to learn hierarchical structure from unstructured input—a feature that is very important for any developmentally plausible model of cognition and to models that seek to explain cortical and biological system organisation and plasticity. The ability to learn structured representations contrasts with current Bayesian models, which assume structured representations a priori or fit them from a specified space of possible representations predefined by the modeller (cf. [42]). Although Bayesian models have a powerful descriptive application, they do not currently offer a mechanistic explanation for how a biological system came to be the way it is.

What are the computational origins of predication, and where does it fall in the taxonomy of cortical computations? What might those origins tell us about why a model of analogy happens to be able to process sentences? Perhaps communicating information across time and space or needing to code for a relationship between representations that goes beyond, or even violates, statistical regularity (such as encountering novel objects and interacting with them, or interacting with old objects in new ways) were challenges that were sufficient to recruit a latent computational mechanism from existing neurocomputational subroutines, in response to the common computational requirement that these problems entailed. One hypothesis is that the underlying computation behind predication is a domain-general abstraction mechanism, such that language processing and analogical reasoning are instances of processing that call upon the same abstraction subroutine, one that might also underlie other “binding”-like phenomena in cognition and perception [13,27], in any neural system that requires relationality or representational hierarchy. An abstraction sub-routine might be a cognitive or computational mechanistic “kind” that is at work in much of human cognition [9,11,22,23,32,43].

Our results suggest a formal and mechanistic synergy between how representational structures are computed, and how energy is expended in both cortical and machine oscillators. The pattern of oscillatory activation observed in the layers of DORA arises directly from the online processing of hierarchical sentence representations—by inductive inference, the cortical signal reflects generation too, rather than “mere” tracking, of hierarchical linguistic representation. Time-based binding via asynchrony, the computational mechanism in our model, links the generation of hierarchical structure to the observable “read-out” in the machine and cortical signal, with broad implications as a computational first principle of cognition in the human brain. Minimally, it explains how a computation gives rise to hierarchical representation *and* why cortical signals stemming from said computation appear the way they do. As such, our results can make the broad prediction that there ought to be temporally dissociable populations oscillating asynchronously; that is, desynchronisation between neural assemblies should occur as a function of the level of linguistic analysis that is being represented in a cortical network at a given time step. In other words, DORA's representational architecture predicts desynchronisation between assemblies oscillating at different frequencies in order to represent, for example, the speech envelope, acoustic phonetic features, syllables/phonemes, morphemes, words, phrases, and sentences. Whether the cross-frequency desynchronisation signal is more strongly observable at stimulus onset and offset or whether representational (de)synchronisation signals should be thought of as the dynamics of phase-to-power cross-frequency coupling over time, as well as a myriad of other important complications, are difficult to predict concretely at this stage. But, it is likely that functional characterisation of the dynamic recruitment or entrainment of cell assemblies during information processing could reveal powerful mechanistic first principles of cortical representation and computation [32,38].

Our results support a view where the organisational principles of cognitive, cortical, and biological systems arise from the nature of the mechanisms that carry out the computations that the system must perform. Our results provide a mechanistic explanation for (1) how our brains form discrete hierarchical representations from holistic unstructured input, as in language comprehension and other domains of cognition, (2) how patterns in cortical oscillations relate to representational structure building, and (3) how the system exploits the fact that time carries information to achieve a representational system that is generative and (de)compositional. The mechanism that gives rise to this state of affairs is time-based binding—the asynchrony of unit firing across layers of the network that allows the model to represent information independently at multiple timescales. The class of possible processing mechanisms that accounts for the output of the cortical computation signal must correspond in some fundamental way to the mechanism through which DORA forms representations. This computational mechanism gives rise to a generative representational hierarchy, as observed in machine and cortical oscillations, serving as an “abstraction engine” for representation in the human brain. Our results suggest that the identification of biological mechanisms, circuits, and subroutines that can perform computations beyond the domain in which they arose, or from which they were derived—a form of computational "recycling"—offers an approach to understanding biological systems, that, through modelling, can derive mechanistic explanations for why systems are the way they are.

## Methods

DORA is a model of how structured relational representations can be learned and represented in a connectionist network. Starting with unstructured representations of objects (i.e., flat feature vectors), DORA learns structured (i.e., predicated) representations of object properties and relations. Importantly, these representations allow DORA to solve a number of problems in analogical and inductive reasoning [19].

For the purposes of the simulations described in this paper, DORA’s learning is not fundamental and so we avoid discussing it further for the purposes of clarity of exposition. It should be noted, however, that all of the propositional structures we describe and use in the simulations can be learned by DORA from scratch. Full details of how these propositions might be learned are given in Doumas et al. [19]. Prior to the simulations, we hardcoded DORA with the Grammatical stimuli from Ding et al., from which it learned propositional sentence structures. For the simulations, we had DORA process stimuli in the different conditions after it had learned and stored sentence structures in its memory.

There are two aspects of DORA’s operation that are fundamental to the current simulations. The first is the manner in which DORA represents propositional structures (sentences) and role-filler bindings (phrases). The second is the manner in which activation spreads from propositions currently in DORA’s focus of attention to propositions in long-term memory (LTM). We describe both of these operations in the sections that follow.

### Representations in DORA

**Fig 7:**
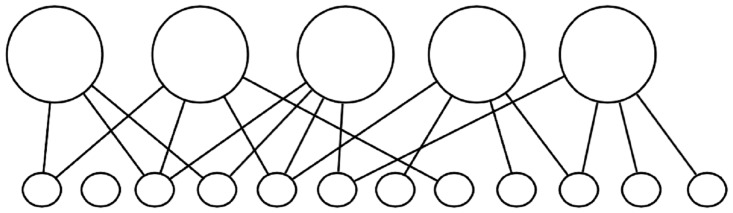
Initial state of the network before learning. The model assumes the existence of objects and features and initially must learn the relationships between features sets and objects. After learning, the model's internal representations are in a predicate calculus (see Figs 1 and 2).

**Fig 8:**
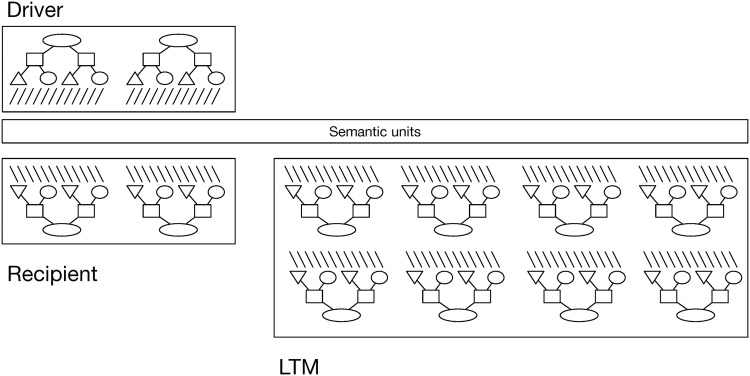
Graphical depiction of banks of units in DORA containing represented propositional structures. The comparison process that is crucial for learning occurs across the driver and recipient (see [19] for details).

In DORA, before learning (although again, not demonstrated here), objects are represented as flat feature vectors (see Fig 7). After learning, relational structures are represented by a hierarchy of distributed and localist codes (see Figs 1, 2 and 8). At the bottom, “semantic” units represent the features of objects and roles in a distributed fashion. At the next level, these distributed representations are connected to localist units (called POs in DORA) representing individual predicates (or roles) and objects; in these simulations, these units represent words. Localist RBs link object and predicate units into role-filler binding pairs for processing; these units represent phrases in these simulations. At the top of the hierarchy, a localist P-unit that represents the sentence links RBs (phrases) into a whole relational proposition or sentence (see Figs 1 and 2).

The token units at the various layers of DORA represent information at a progressively more conjunctive level. While independence of roles and filler is maintained in the semantic and PO units, RB units code conjunctively for specific phrases or role-filler bindings, and P-units code for conjunctions of role-filler sets into full relational propositions, or sentences in this case. Conjunctive binding is sufficient for long-term storage but violates role-filler independence and so fails fundamentally for any tasks that require independent representation of roles and fillers [20,44], as processing hierarchical and symbolic structure requires that representational elements in a system can be composed into structures in a manner that does not violate the independence of those elements (see [26,40]). Consequently, during structured processing, DORA must maintain binding information in those units (semantics and POs) that maintain role-filler independence.

DORA is composed of a number of sets or banks of units (see Fig 8). The driver is the current focus of attention, or what DORA is thinking about, at any given moment. The recipient is a set of units representing propositions that are available for comparison with the propositions in the driver. Hummel and Holyoak [45] have described the recipient in terms of Cowan’s [46] active memory. Finally, LTM is the set of propositions that DORA has encountered in the past that are not currently active. All banks of units are connected via a common pool of semantic units. During processing, activation flows from units in the driver, to the semantic units, and then to units in the recipient and LTM. The flow of activation from driver to recipient is fundamental for a number of DORA’s operations, including analogical mapping, inference, and predicate learning and refinement. The flow of activation from driver to LTM is important for retrieval. None of the current simulations rely on operations involving flow of activation between driver and recipient, so we do not discuss the recipient set any further.

The driver, as the focus of DORA’s attention, is the starting point for all of DORA’s processing. When DORA performs any structured processing, role-filler bindings must be maintained in the units that maintain role-filler independence (see above). This dynamic binding (bindings that do not violate role-filler independence and can be created and destroyed on the fly [20]) information is carried in DORA using time. Specifically, DORA uses systematic asynchrony of firing to maintain role-filler bindings.

In DORA, binding information can be carried either by synchrony (as in the symbolic-connectionist model Learning and Inference with Schemas and Analogies [LISA] [21]) or by systematic asynchrony of firing, with bound role-filler pairs firing in direct sequence. Asynchrony-based binding, or what we call "time-based binding," allows roles and fillers to be coded by the same pool of semantic units, which allows DORA to learn representations of relations from representations of objects (Doumas et al. [19]). During asynchronous binding, in which a proposition like *waste* (games, time) becomes active in the driver (see Fig 8), the units representing *waster* fire (along with units conjunctively coding for *waster*+games and for the *waste* [games, time] proposition; see Fig 1A), followed directly by the units representing games (along with units conjunctively coding for *waster*+games and for the *waste* [games, time] proposition; see Fig 1B), representing the binding of *waster* to games.

Then, the units representing *wasted* fire (along with units conjunctively coding for *wasted*+time and for the *waste* (game, time) proposition; see Fig 1C), followed directly by the units representing time (along with units conjunctively coding for *wasted*+time and for the *waste* [time, games] proposition; see Fig 1D), representing the binding of *wasted* to time. In short, bound role-filler pairs fire in direct sequence and out of synchrony with other bound role-filler pairs. These patterns of sequential oscillation dynamically code role-filler bindings in DORA and underlie DORA’s capacity to use the representations that it learns to support relational reasoning (e.g., analogical mapping, schema induction, and relational induction; see [19]) and to learn structured relational representations from unstructured object representations.

While establishing time-sharing patterns of firing in a connectionist model might seem complicated, as demonstrated by Hummel and Holyoak [21,45] and Doumas et al. [19], it is actually rather simple. In DORA, each token unit is actually a coupling of two units, an exciter and a yoked inhibitor. The exciter unit behaves like a conventional node in a neural network, taking and passing input to units at higher and lower levels and laterally inhibiting and being inhibited by units in the same layer. Each exciter is also yoked to an inhibitor unit that integrates input over time and, when a threshold is reached, forces the yoked exciter unit to inactivity, allowing other units to become active.

Continuing the above example, when the phrase/RB unit coding for *waster*+games fires, the two word/PO units connected to that phrase/RB—*waster* and games—compete to become active. Due to noise in the system, one of these tokens will become slightly more active and inhibit the other to inactivity. For example, *waster* might become slightly more active and inhibit games to inactivity. After some time firing, *waster*’s inhibitor unit will fire, forcing it to inactivity and allowing the word/PO representing games to fire. Similarly, after some time firing, the inhibitor yoked to the phrase/RB unit coding *waster*+games will fire, forcing that unit to inactivity and allowing another phrase/RB (e.g., *wasted*+time) to fire. Establishing the pattern of firing described above requires units in different layers firing at different timescales. This pattern can be achieved in any number of ways, the simplest being setting the threshold of the inhibitor units appropriately (e.g., word/PO inhibitors have half the firing threshold of phrase/RB inhibitors). In DORA, inhibitor units of words/POs integrate input both from their yoked exciter and from the exciters of all units in the above layers (e.g., phrases/RBs). Consequently, words/POs naturally oscillate at twice the frequency of phrases/RBs.

Crucially, sequential firing of related constituent elements is a necessary property of binding via synchrony and systematic asynchrony. When DORA performs any structured processing, a pattern will invariably emerge wherein bound elements within a larger compositional proposition will fire in direct sequence and at a different timescale than units coding for conjunctions of independently bound elements and full propositional compounds. In the following section, we show that the pattern of activation produced by DORA as it processes compositional structures very closely matches the temporal pattern of spike activity observed in Ding et al. [6] when people process sentences.

### Simulation of Ding et al. [6]

We simulated the Ding et al. [6] studies using the same English sentences used in their Experiments 5 and 6 (with native English speakers). All of these sentences took the form modifier-noun-verb-noun, forming sentences like “new plans give hope,” “fun games waste time,” and “dry fur rubs skin.” DORA can represent hierarchical propositions by representing propositional structures as arguments of other propositional structures. For example, to represent “dry fur rubs skin,” the modified noun phrase “dry fur” can be represented explicitly by the propositional structure *dry* (fur), which can then serve as the argument of the agent role of the *rubs* relation (see Fig 2; details of higher-order structure representation in LISA, from which DORA is descended, and from DORA can be found in Hummel & Holyoak [21] and in Doumas et al. [19] respectively).

To simulate Ding et al.’s main experimental procedure, we allowed DORA to process Ding et al.’s English sentences one at a time, using the representations of those sentences. Representations of the sentence structures (e.g., Figs 1 and 2) entered the driver (i.e., were attended to). We then activated these sentence structures one word at a time. That is, we activated the semantic units encoding the first word for 110 iterations, then the second word for 110 iterations, and so forth. As semantic units became active, they passed activation to token units in the driver. The units in the driver responded to the pattern of firing in the semantic units (i.e., the units fired to represent and encode binding information; see Fig 8).

To simulate Ding et al.’s control condition we allowed DORA to process Ding et al.’s random word sequences one at a time. In this *Word List* condition, there were no syntactic relationships between words. Representations of the sentence word sequence entered the driver (i.e., were attended to). DORA processed the word as it normally would (i.e., the units coding each term fired, but because the sequence of words included no propositional structure, only word/PO units fired during processing). We tracked firing rate of all the nodes in the driver as DORA processed the sentences. The results of the simulation and the comparison to the patterns observed by Ding et al. are presented in Fig 3. Interestingly, the pattern of firing of the nodes in the various layers of DORA very closely mirror the patterns observed by Ding et al. Specifically, just like the human participants, DORA showed an activation burst only at the rate of word representation, or four times the rate of the whole sentence burst (i.e., the word/PO units firing in the 4 Hz range).

### Retrieval and activation of LTM

DORA, like LISA, performs memory retrieval by firing propositions in the driver and allowing activation to flow to LTM via the shared semantic units. Units in LTM respond to the pattern of activation in the semantic units imposed by the units in the driver and are retrieved into active memory (the recipient) via a Luce [47] choice function (see Doumas et al. [19]). For example, when DORA is "thinking about" how games waste time, and the proposition *wastes* (games, time) becomes active in the driver, activation will flow through the semantic units to units in LTM that share semantic overlap with the word/PO units becoming active in the driver (i.e., *wasters*, *wasted-things*, games, time, and things like them). We used this property of the model to further test whether the model is representing syntactic structure. We had DORA to process versions of Ding et al.’s word sequences in the Word List condition (please see S1 Text), and we created a *Jabberwocky* condition where there were only syntactic relationships between words but no typical compositional semantic relationships. Representations of the sentence word sequence entered the driver (i.e., were attended to). DORA processed the sentences one word at a time (i.e., the units fired to represent and encode binding information, as above). We tracked firing rate of all the nodes in the driver as DORA processed the sentences. The results of the simulation and the comparison to the patterns observed in the experimental conditions are in Figs 3, 4, 5 and 6. Ding et al. also manipulated a form of constituency—the linguistic relationship between discrete units and larger units, in this case, between syllables and words—to determine if there was evidence for cortical tracking of these various units. They found that words with multiple syllables elicited oscillations that tracked with the duration of the phrase boundary, not just syllables, which were fixed at 250 ms or 4 Hz. Ding et al. found 2 Hz and 4 Hz activity for two disyllabic words that together formed a phrase (a stimulus stream of (xx)(xx), but no 1 Hz activity. Since DORA does not have the perceptual apparatus to process auditory signals, we created a text analogue of the phrase condition (Phrases condition).

### Simulations in a RNN

**Fig 9:**
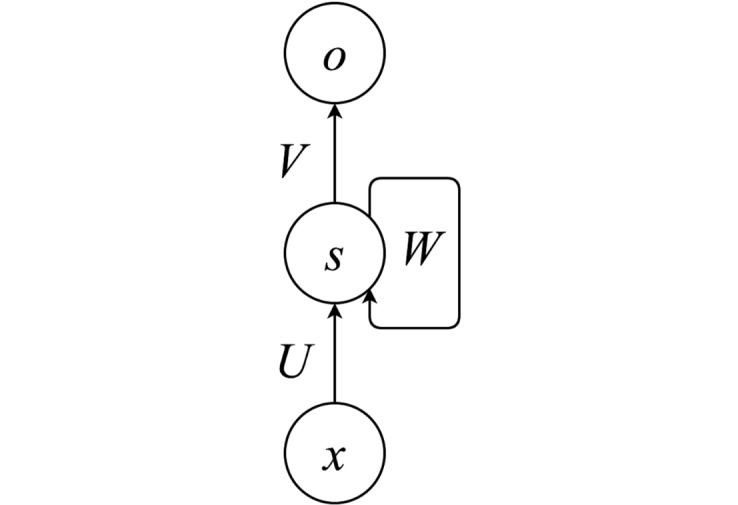
Architecture of the three-layer RNN. *x*, *s*, and *o* represent vectors of activation of units in the input, recurrent, and output layers, respectively. *U*, *V*, and *W* represent input, output, and recurrent weight matrices, respectively.

To test whether the oscillatory pattern observed by Ding et al. and in our DORA simulations can be observed in a system without time-based binding and without representational hierarchy, we repeated all four simulations in a RNN implemented in Theano [48]. The network had one hidden layer (see Fig 9).

#### Sentence and word coding

In order to train the RNNs in the current simulations, two encoding systems were used. In the input encoding system, each word was assigned a randomly generated vector of zeros and ones of size ten. One additional 1-valued vector of size ten was used to represent the beginning of any sentence.

In the output encoding system, a localist representation was used instead. For this, each word was assigned a randomly chosen one-hot vector of size *n* = 333, where 333 is the number of different words across datasets plus one word for the end of the sentence marker. In this system, then, unit *i* = 1 represents word *i*, and the rest of the units are zeros.

#### Network architecture

The RNN used for the simulations described here consisted of three layers of units: input, recurrent, and output (see Fig 9). Input layers have ten units. Output layers have 333 units, one unit per word in the dataset plus one extra unit for the “end of the sentence” word. Recurrent layers have (number of words per phrase * ten) units. The network processed the input sequence over *t* (number of words per phrase) time steps, receiving one word per time step. The input layer activation at time step *t* corresponds to the random generated vector of zeros and ones of size ten that was associated with the word in the input encoding scheme. The activation in the recurrent and output layers of the RNN is calculated according to:

$$s_{t}=\text{tanh}(Ux_{t}+Ws_{t-1})$$

$$o_{t}=softmax(Vs_{t})$$

where *s*_*t*_ and *o*_*t*_ are the activation vectors at time step t of the recurrent and output layers; *U*, *V*, and *W* are the input, recurrent, and output weight matrices. The softmax function $f\left(z_{i}\right)=\;\frac{e^{z_{i}}}{\sum_{j}e^{z_{j}}}$ results in a vector of activations that sum to one and can be interpreted as probabilities. Since a localist representation was used in the output layer, each element *i* of *o*_*t*_ is the network’s estimated probability that word *i* will be the input at time step *t* + 1.

#### Network training

For each condition (Grammatical, Jabberwocky, Word List, Phrases), the RNN was presented with the sentences, one word by time step, and was trained to predict the next word in the current sentence. The prediction of the network was taken to be the index of the output unit with maximum activation.

For all conditions, the input sentences always had the form (“beginning” “word_1” “word_2”…“word_n”), while the criterion sentences always had the form (“word_1” “word_2”…”word_n” “end”). Since the first word of the sentence could not be predicted from the beginning vector, the recurrent state activations *s* resulting from feeding the beginning vector to the RNNs were discarded and are not discussed further.

The RNNs were trained with gradient descent and adaptive learning rate. The learning rate decreased to half its magnitude every time the cost increased (the initial learning rate was set to 0.005 for all conditions except for the Only NPs condition in which it was set to 0.01). All RNNs were trained for 150 epochs, at which point the network achieved perfect classification. The loss function used for training was cross-entropy.

## Supporting information

S1 Text Stimuli. (DOCX)

## Abbreviations

adj: adjective
AdjP: adjective phrase
DORA: Discovery of Relations by Analogy
IP: inflectional phrase or sentence
LISA: Learning and Inference with Schemas and Analogies
NLP: natural language processing
n: noun
NP: noun phrase
PO: propositional object
P-unit: proposition node
RB unit: role-filler binding node
RNN: recurrent neural network
v: verb
VP: verb phrase

## References

[1] Belin P, Zatorre RJ, Lafaille P, Ahad P, Pike B. Voice-selective areas in human auditory cortex. Nature. 2000 1 20;403(6767):309–12. 10.1038/35002078 10659849

[2] Luo H, Poeppel D. Phase patterns of neuronal responses reliably discriminate speech in human auditory cortex. Neuron. 2007 6 21;54(6):1001–10. 10.1016/j.neuron.2007.06.004 17582338 PMC2703451

[3] Giraud AL, Poeppel D. Cortical oscillations and speech processing: emerging computational principles and operations. Nat Neurosci. 2012 4 1;15(4):511–7. 10.1038/nn.3063 22426255 PMC4461038

[4] Ghitza O. Linking speech perception and neurophysiology: speech decoding guided by cascaded oscillators locked to the input rhythm. Front Psychol. 2 (2011): 130. 21743809 10.3389/fpsyg.2011.00130 PMC3127251

[5] Peelle JE, Davis MH. Neural oscillations carry speech rhythm through to comprehension. Front Psychol. 2012 9 6;3:320 10.3389/fpsyg.2012.00320 22973251 PMC3434440

[6] Ding N, Melloni L, Zhang H, Tian X, Poeppel D. Cortical tracking of hierarchical linguistic structures in connected speech. Nat Neurosci. 2016 1 1;19(1):158–64. 10.1038/nn.4186 26642090 PMC4809195

[7] Giraud AL, Kleinschmidt A, Poeppel D, Lund TE, Frackowiak RS, Laufs H. Endogenous cortical rhythms determine cerebral specialization for speech perception and production. Neuron. 2007 12 20;56(6):1127–34. 10.1016/j.neuron.2007.09.038 18093532

[8] Kiebel SJ, Daunizeau J, Friston KJ. A hierarchy of time-scales and the brain. PLoS Comput Biol. 2008 11 14;4(11):e1000209 10.1371/journal.pcbi.1000209 19008936 PMC2568860

[9] Poeppel D. The neuroanatomic and neurophysiological infrastructure for speech and language. Curr Opin Neurobiol. 2014 10 31;28:142–9. 10.1016/j.conb.2014.07.005 25064048 PMC4177440

[10] Barsalou LW. Perceptions of perceptual symbols. Behav Brain Sci. 1999 8 1;22(04):637–60. 10.1017/s0140525x99002149 11301525

[11] Gallistel CR. The organization of learning. The MIT Press; 1990.

[12] Pinker S, Jackendoff R. The faculty of language: what's special about it? Cognition. 2005 3 31;95(2):201–36. 10.1016/j.cognition.2004.08.004 15694646

[13] Von der Malsburg C. The what and why of binding: the modeler’s perspective. Neuron. 1999 9 30;24(1):95–104. 10677030 10.1016/s0896-6273(00)80825-9

[14] Berwick RC, Friederici AD, Chomsky N, Bolhuis JJ. Evolution, brain, and the nature of language. Trends Cogn Sci. 2013 2 28;17(2):89–98. 10.1016/j.tics.2012.12.002 23313359

[15] Chomsky N. Syntactic structures. Walter de Gruyter; 1957.

[16] Halle M. Phonology in generative grammar. Word. 1962 1 1;18(1–3):54–72.

[17] Poeppel D, Idsardi WJ, Van Wassenhove V. Speech perception at the interface of neurobiology and linguistics. Phil Trans R Soc B-Biol Sci. 2008 3 12;363(1493):1071–86. 10.1098/rstb.2007.2160 PMC2606797 17890189

[18] Martin AE. Language processing as cue integration: Grounding the psychology of language in perception and neurophysiology. Front Psychol. 2016;7. 10.3389/fpsyg.2016.00120 PMC4754405 26909051

[19] Doumas LA, Hummel JE, Sandhofer CM. A theory of the discovery and predication of relational concepts. Psychol Rev. 2008 1;115(1):1 10.1037/0033-295X.115.1.1 18211183

[20] Doumas LA, Hummel JE. Approaches to modeling human mental representations: What works, what doesn’t and why The Cambridge handbook of thinking and reasoning, ed. Holyoak KJ & Morrison RG. 2005 4 18:73–94.

[21] Hummel JE, Holyoak KJ. Distributed representations of structure: A theory of analogical access and mapping. Psychol Rev. 1997 7;104(3):427.

[22] Park HJ, Friston K. Structural and functional brain networks: from connections to cognition. *Science*. 2013, 342 Jg., Nr. 6158, S. 1238411. 10.1126/science.1238411 24179229

[23] Gallistel CR, King AP. Memory and the computational brain: Why cognitive science will transform neuroscience. John Wiley & Sons; 2011.

[24] Pylkkänen L., Bemis D. K., & Elorrieta E. B. (2014). Building phrases in language production: An MEG study of simple composition. Cognition, 133(2), 371–384. 10.1016/j.cognition.2014.07.001 25128795

[25] Pinker S, Prince A. On language and connectionism: Analysis of a parallel distributed processing model of language acquisition. Cognition. 1988 3 1;28(1–2):73–193. 2450717 10.1016/0010-0277(88)90032-7

[26] Russell SJ, Norvig P, Canny JF, Malik JM, Edwards DD. Artificial intelligence: a modern approach. Upper Saddle River: Prentice hall; 2003 1.

[27] Treisman A. The binding problem. Curr Opin Neurobiol. 1996 4 30;6(2):171–8. 8725958 10.1016/s0959-4388(96)80070-5

[28] Hebb DO. The organization of behavior: A neuropsychological approach. John Wiley & Sons; 1949.

[29] Shastri L, Ajjanagadde V. From simple associations to systematic reasoning: A connectionist representation of rules, variables and dynamic bindings using temporal synchrony. Behav Brain Sci. 1993 9 1;16(03):417–51.

[30] Marcus GF. Rethinking eliminative connectionism. Cogn Psychol. 1998 12 31;37(3):243–82. 10.1006/cogp.1998.0694 9892549

[31] Penn DC, Holyoak KJ, Povinelli DJ. Darwin's mistake: Explaining the discontinuity between human and nonhuman minds. Behav Brain Sci. 2008 4 1;31(02):109–30. 18479531 10.1017/S0140525X08003543

[32] Buzsáki G. Neural syntax: cell assemblies, synapsembles, and readers. Neuron. 2010 11 4;68(3):362–85. 10.1016/j.neuron.2010.09.023 21040841 PMC3005627

[33] Doumas LA, Hummel JE. Computational models of higher cognition The Oxford Handbook of Thinking and Reasoning. 2012 4 19:52–66.

[34] Doumas LA, Hummel JE. A computational account of the development of the generalization of shape information. Cogn Sci. 2010 5 1;34(4):698–712. 10.1111/j.1551-6709.2010.01103.x 21564231

[35] Lim A, Doumas LA, Sinnett S. Supramodal representations in melodic perception. In 36th Annual Conference of the Cognitive Science Society, Quebec, Canada 2014.

[36] Morrison RG, Doumas LA, Richland LE. A computational account of children’s analogical reasoning: balancing inhibitory control in working memory and relational representation. Dev Sci. 2011 5 1;14(3):516–29. 10.1111/j.1467-7687.2010.00999.x 21477191

[37] Sandhofer CM, Doumas LA. Order of presentation effects in learning color categories. J Cogn Dev. 2008 4 30;9(2):194–221.

[38] Hanslmayr S, Staresina BP, Bowman H. Oscillations and Episodic Memory: Addressing the Synchronization/Desynchronization Conundrum. Trends Neurosci. 2016 1 31;39(1):16–25. 10.1016/j.tins.2015.11.004 26763659 PMC4819444

[39] Heim I, Kratzer A. Semantics in generative grammar. Oxford: Blackwell; 1998 1 2.

[40] Markman AB. Knowledge representation. Psychology Press; 1999.

[41] Hinton GE, Osindero S, Teh YW. A fast learning algorithm for deep belief nets. Neural Comput. 2006 7;18(7):1527–54. 10.1162/neco.2006.18.7.1527 16764513

[42] Tenenbaum JB, Kemp C, Griffiths TL, Goodman ND. How to grow a mind: Statistics, structure, and abstraction. Science. 2011 3 11;331(6022):1279–85. 10.1126/science.1192788 21393536

[43] Poeppel D. The maps problem and the mapping problem: two challenges for a cognitive neuroscience of speech and language. Cogn Neuropsychol. 2012 3 1;29(1–2):34–55. 10.1080/02643294.2012.710600 23017085 PMC3498052

[44] Doumas LA, Hummel JE. Comparison and mapping facilitate relation discovery and predication. PLoS ONE. 2013 6 25;8(6):e63889 10.1371/journal.pone.0063889 23825521 PMC3692476

[45] Hummel JE, Holyoak KJ. A symbolic-connectionist theory of relational inference and generalization. Psychol Rev. 2003 4;110(2):220 12747523 10.1037/0033-295x.110.2.220

[46] Cowan N. Metatheory of storage capacity limits. Behav Brain Sci. 2001 2 1;24(01):154–76. 10.1017/s0140525x01003922 11515286

[47] Luce RD. On the possible psychophysical laws. Psychol Rev. 1959 3;66(2):81 13645853 10.1037/h0043178

[48] Bergstra J, Breuleux O, Bastien F, Lamblin P, Pascanu R, Desjardins G, Turian J, Warde-Farley D, Bengio Y. Theano: A CPU and GPU math compiler in Python. InProc. 9th Python in Science Conf 2010 Jun (pp. 1–7).

