# Supplementary material for: A mechanism for the cortical computation of hierarchical linguistic structure
Source: PLoS Biol. 2017 Mar 2;15(3):e2000663. doi: 10.1371/journal.pbio.2000663 (PMC5333798; doi:10.1371/journal.pbio.2000663)
Supplement: S1 Text — (DOCX) [file pbio.2000663.s001.docx]

APPENDIX

**List of stimuli**

Grammatical condition (please see Supplementary Table 1 of Ding et al., 2016 )

*Jabberwocky condition*

adj n v n

fat shelf drove light

wood girls shine skin

tan lamps rubs eggs

gold fur stole steak

dry fox cook notes

sly chefs wrote trees

top boss plant kids

our teams love hope

two moms give nests

all plans built bugs

new ants hunt dogs

large apes claw tea

teen cats brewed time

rude cooks waste girls

rich games hurt ships

fun toys tied you

pink waves hear shoes

huge ears hit hearts

deaf aunt hide hate

his words ate blood

kind fight leak holes

long sharks dig jeans

dead dogs like tests

smart kids fail cups

slim boys warm bread

sick doors make town

rear hands fill chefs

pale smells smack grass

bad foes caused food

mad lamb brings flies

quiet fork miss stars

soft frogs show camp

green skies flee hills

black beer climb cents

tall goat costs speech

grey guys gave tears

iced kings shed gas

old eyes need doors

blue cars closed plates

white child hang cars

young threads sold milk

thin store drink cows

their cubs lost cheese

cute farms cuts germs

six knife killed mom

sharp soap scared hats

round sound wear walls

loud clowns paint streets

weird sons cross hums

her bears sang worms

giant dudes caught space

drunk bags take rooms

little chick cleaned roads

brown maids block farms

four rocks ruined snow

big flood melts tires

fierce ground slash bills

warm blades paid fear

keen friends sensed cans

poor rat holds trucks

*Word List condition*

*cute wood tan gold*

*shelf girls lamps fur*

*drove shine rubs stole*

*light skin eggs steak*

*dry sly top our*

*fox chefs boss teams*

*cook wrote plant love*

*notes trees kids hope*

*two all new large*

*moms plans ants apes*

*give built hunt claw*

*nests bugs dogs tea*

*teen rude rich fun*

*cats cooks games toys*

*brewed waste hurt hit*

*time girls ships you*

*pink huge deaf his*

*waves ears aunt words*

*hear tied warm caused*

*shoes hearts hate blood*

*kind long dead smart*

*fight sharks dogs kids*

*leak dig like fail*

*holes jeans tests cups*

*slim sick rear pale*

*boys doors hands smells*

*hide make fill smack*

*bread town chefs grass*

*bad mad quiet soft*

*foes lamb fork frogs*

*ate brings miss show*

*food flies stars camp*

*green black tall gray*

*skies guys goat beer*

*flee climb costs gave*

*hills cents speech tears*

*iced old blue white*

*kings eyes cars child*

*shed need closed hang*

*gas doors plates cars*

*young thin their cute*

*threads store cubs farms*

*sold drink lost cuts*

*milk cows cheese germs*

*six sharp round loud*

*knife soap sound clowns*

*killed scared wear paint*

*mom hats walls streets*

*weird her giant drunk*

*sons bears dudes chick*

*cross sang caught take*

*hums worms space rooms*

*little brown four big*

*bags maids rocks flood*

*cleaned block ruined melts*

*roads farms snow tires*

*fierce warm keen poor*

*ground blades friends rat*

*slash paid sensed holds*

*bills fear cans truck*

Phrase condition

fat rat wood shelf

tan girls gold lamps

dry fur sly fox

top chefs our boss

two teams all moms

new plans large ants

teen apes rude cats

rich cooks fun games

pink toys huge waves

healthy ears his aunt

kind words long fight

dead sharks smart dogs

slim kids sick boys

rear doors pale hands

bad smells mad foes

quiet lamb smooth fork

green frogs black skies

tall guys grey goat

iced beer old kings

blue eyes white cars

young child thin threads

their store cute cubs

six farms sharp knife

round soap loud sound

weird clowns her sons

giant bears drunk dudes

little chick brown bags

four maids big rocks

fierce flood warm ground

keen blades poor friend

**References**

Ding N, Melloni L, Zhang H, Tian X, Poeppel D. Cortical tracking of hierarchical linguistic structures in connected speech. Nat Neurosci. 2016 Jan 1;19(1):158-64.
